# Neurotoxicity of Silver Nanoparticles and Non-Linear Development of Adaptive Homeostasis with Age

**DOI:** 10.3390/mi14050984

**Published:** 2023-04-30

**Authors:** Anna A. Antsiferova, Marina Yu. Kopaeva, Vyacheslav N. Kochkin, Alexander A. Reshetnikov, Pavel K. Kashkarov

**Affiliations:** 1 National Research Center “Kurchatov Institute”, Akademika Kurchatova sq., 123182 Moscow, Russia; m.kopaeva@mail.ru (M.Y.K.); kochkin_vn@nrcki.ru (V.N.K.); reshetnikov_aal@nrcki.ru (A.A.R.); kashkarov_pk@nrcki.ru (P.K.K.); 2 Moscow Institute of Physics and Technologies, Institutskii Lane, Moscow Region, 141700 Dolgoprudny, Russia; 3 Department of Physics, Lomonosov Moscow State University, GSP-1, Leninskiye Gory, 119991 Moscow, Russia

**Keywords:** nanoparticle, adaptive homeostasis, silver nanoparticles, ageing, behavioral functions, anxiety, individual content, mice, neutron activation analysis, bioaccumulation

## Abstract

For the first time in the world, the behavioral functions of laboratory mammals exposed to silver nanoparticles were studied with regard to age. Silver nanoparticles coated with polyvinylpyrrolidone with a size of 8.7 nm were used in the present research as a potential xenobiotic. Elder mice adapted to the xenobiotic better than the younger animals. Younger animals demonstrated more drastic anxiety than the elder ones. A hormetic effect of the xenobiotic in elder animals was observed. Thus, it is concluded that adaptive homeostasis non-linearly changes with age increase. Presumably, it may improve during the prime of life and start to decline just after a certain stage. This work demonstrates that age growth is not directly conjugated with the organism fading and pathology development. Oppositely, vitality and resistance to xenobiotics may even improve with age at least until the prime of life.

## 1. Introduction

**Figure 1:**
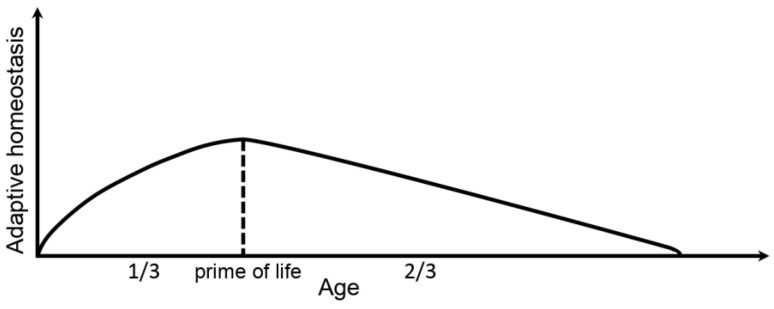
Adaptive homeostasis development with age during the whole maximal lifespan.

Adaptive homeostasis by definition is a transient expansion or contraction of the homeostatic range for any given physiological parameter in response to exposure to sub-toxic, non-damaging, signaling molecules or events, or the removal or cessation of such molecules or events [1]. In other words, adaptive homeostasis is a capability of an organism to adapt to a damaging factor, for instance, to toxic compounds. It is assumed that adaptive homeostasis decreases in a direct ratio to age increase [2]. However, this is a rather questionable point. Other researchers believe that the blossom of vitality falls on the first 1/3 of the maximal lifespan, i.e., middle age, which is followed by a gradual decline of physiological functions [3]. Therefore, presumably, adaptive homeostasis may also improve until about the first 1/3 of the lifespan and fade out later (Figure 1). Importantly, Figure 1 demonstrates the total lifespan not as an average but as a maximum life expectancy. Thus, the ‘prime of life’ point corresponds to 42–45 years old for a human. 

Presently, silver nanoparticles (AgNPs) are one of the most sought-after commercial nanotechnology products in the market [4]. They are widely used in the medicine, pharmaceutics, food, cosmetic, and light industries due to their excellent antiseptic properties [5,6,7,8]. At the same time, AgNPs may cause toxicity in healthy tissues as well. AgNPs sized smaller or larger than 10 nm could both cause neuronal cell death after entering the brain. It was shown that inflammation and increased oxidative stress followed by apoptosis are likely to be the main mechanisms of AgNPs toxicity [9,10]. AgNPs demonstrated toxicity in the testes after six months of oral exposure to Sprague Dawley rats [11]. Also, AgNPs had cytotoxic effects on the HepG2 cell line and primary liver cells of mice in in vitro study [12]. There is a large number of studies confirming AgNPs toxicity in regard to different cell types and tissues in in vitro and in vivo experiments. There is no doubt that AgNPs may be considered as a xenobiotic for humans and animals in certain dosages. 

AgNPs demonstrate special bioaccumulative properties. They can accumulate in different tissues of an organism, particularly, in the brain [13,14,15,16], and disturb cognitive and behavioral functions [17,18,19,20], which manifested in violation of the formation and consolidation of memory and learning, a decrease in social behavior and locomotor activity, and anxiety increase. Thus, we have shown before that the behavioral and cognitive functions of laboratory mice exposed to AgNPs demonstrated 3-staged changes such as anxiety increase at first, development of adaptation mechanism, manifested in the increase of exploration behavior, and finally disturbance of the long-term contextual memory [17]. It is clear that the widespread use of AgNPs may be not safe enough and even harmful to humans and the environment. Uncontrolled pollution of the environment with AgNPs may potentially lead to the disappearance of some links of a food chain, followed by the disturbance of ecosystems.

The long-term effects of AgNPs are not well studied due to the certain ethical restrictions for the experiment duration accepted worldwide and the relative novelty of Nanosafety field. However, some of them showed long-term contextual memory impairment in mice followed by prolonged oral exposure to AgNPs [17] and enhancement of the oxidative stress in the cerebral myelin presumably caused by the ultrastructural disturbances in myelin sheaths after 28 days of oral exposure of rats to AgNPs [21]. A significant decrease in testosterone levels, an increase in luteinizing hormone levels, a decrease in superoxide dismutase activity, an increase in malondialdehyde levels, and a decrease in sperm viability were found in Sprague Dawley rats after 6 months of AgNPs oral exposure [22]. In general, the ‘long-term effects’ term has a double sense. On the one hand, these are the effects caused by the long-term administration of the studied chemical compound. On the other, these are the effects appearing in the late period of a lifespan. Thus, negative effects in Drosophila due to AgNPs exposure during the early period of the lifespan appeared mostly in the late period of it [23]. Such effects caused by AgNPs exposure in the late period of life are not known in mammals. 

The most frequently discussed mechanism of AgNPs’ toxic action is considered to be oxidative stress [24]. Oxidative stress is also regarded as the reason for neurodegenerative disease development. Thus, AgNPs may potentially increase the risk of Alzheimer’s and Parkinson’s diseases [25]. However, another work demonstrates AgNPs’ anti-inflammatory properties and discusses the potential of their application against chronic neurodegeneration [26]. 

The toxic effects of AgNPs on living organisms with regard to age have not been well studied. However, there is a sole work where dietary exposure of Drosophila to 12.5 nm AgNPs during early life with regard to age as well as AgNPs mechanisms of toxicity are investigated [23]. It was shown that exposure to AgNPs triggers multiple adverse effects on functional aging, including shortened lifespan, age-dependent decline, and loss of stress resistance and intestinal integrity. AgNPs inactivate antioxidant pathways in old—but not young—animals, increasing susceptibility to ROS in old age. Mammalian lifespan is longer and more complex than that of insects. Therefore, it is necessary to conduct such studies with model mammals to extrapolate the data to humans. Thus, we investigated the influence of AgNPs on the behavioral functions of mammals at different ages as well as their accumulation in different tissues. This work’s general objective was to determine the direction of the adaptive homeostasis vector change. Does it decline with age growth or non-linearly change?

## 2. Materials and Methods

A food supplement, Argovit S (Vector-Vita, Novosibirsk, Russia), recommended for GUT disease treatment in veterinary [27], was used as the AgNPs. The nanoparticles were coated with polyvinylpyrrolidone. Polivinylpyrrolidone is a hydrophilic polymer that forms hydrogen bonds with water molecules and provides solubility and stability of the AgNPs in water media. The size and stability of the AgNPs were studied by dynamical light scattering (DLS) (Malvern Zetasizer Nano ZS, Malvern, UK). The shape and chemical composition of the AgNPs were studied by transmission electron microscopy (TEM) (Thermo Fisher Scientific, Waltham, MA, USA) with energy-dispersive X-ray spectroscopy (EDX). 

Male mice C57BL/6 obtained from the “Stolbovaya” branch of the Federal Medical Biological Agency of Russia were used as a mammalian model. The behavioral functions of the animals were studied in the open field (OF), elevated plus maze (EPM), and light-dark box (LDB). The silver content in animal organs was measured by means of instrumental neutron activation analysis (INAA) with the application of research nuclear reactor IR-8 (Moscow, Russia) with a capacity of 8 MWth and a gamma spectrometer (ORTEC, Oak Ridge, TN, USA).

## 3. Experiment Scheme

### 3.1. Study of Nanoparticle’s Morphology

The optimal concentration of AgNPs solution was selected to prevent multiparticle scattering and to ensure a sufficient counting time. The original 10 mg/mL solution was diluted 10-, 50-, and 100-fold with deionized Milli-Q water (MilliporeSigma, Burlington, MA, USA). The optimal concentration was established as 0.1 mg/mL on the base of the empirical research. This concentration was further used to study the AgNPs size. An amount of 10 mL of the initial solution was poured into a container, which was hermetically sealed then and kept in the dark at +2 °C for 1 year to study the nanoparticle’s stability. A concentration of 0.2 mg/mL was applied for AgNPs visualization by TEM and EDX measurements. 

### 3.2. Animal Treatment and Preparation Administration

All the procedures with animals were conducted according to the rules of the Ministry of Health of the Russian Federation (No 267 from 19.06.2013) and approved by the Local Ethics Committee for Biomedical Research of the National Research Center “Kurchatov Institute” (No 01 from 10.02.2017). 

The mice were kept in individual cages during the whole experiment with unlimited access to food and water in rooms with an automatically maintained temperature of 23 ± 2 °C and a 12/12-h day/night cycle. The room’s humidity was controlled at 45 ± 10%. The animal’s body mass was controlled weekly.

There were two groups of mice 3 months apart in age. The mice in the first group were introduced into the experiment at the age of 2 months (younger, control 1, n = 20). The mice in the second group were introduced into the experiment at the age of 5 months (elder, control 2, n = 24). Both groups were divided into control (control 1, n =10; control 2, n = 12) and experimental subgroups (younger, n = 10; elder, n = 12). 

AgNPs were introduced daily orally with drinking water in the amount of 50 µg per day per animal for 60 days. The drinkers were weighed weekly to control the consumed amount of liquid. It was noticed that mice consumed an equal amount of liquid (3.8 mL per day) at constant air humidity. Based on this, the required amount of AgNPs was dissolved in the pure water Osmoteck 40-3-2 (OOO “Pharmsystemy”, Besedy, Moscow region, Russia).

The mice were about 5 (younger) and 8 (elder) months old by the testing time points and the end of the experiment. Conditionally assuming a maximum mouse lifespan of over 30 months (no less than 30 months) [28], it can be easily calculated that the prime of life point (Figure 1) is 10 months. Thus, according to the second theory considered in the introduction, an improvement of adaptive homeostasis should take place between the observed ages. According to the first theory, a gradual decline of adaptive homeostasis would be observed within the considered period of mice’s life.

### 3.3. Behavioral Tests

Behavioral tests for locomotor activity, exploration behavior, and anxiety assessment started on the 61st day of the experiment. At the same time, we continued the experimental animal exposure to AgNPs in order to exclude possible elimination processes. EthoVision XT 8.5 (Noldus Information Technology, Wageningen, The Netherlands) video-tracking equipment and software were used for detecting the animal’s position and movement.

#### 3.3.1. Open Field

Locomotor activity, anxiety, and exploration behavior were estimated in the OF on the 61st day of the experiment similarly to [17]. Briefly, a mouse was placed in the middle of a plastic circular arena (d = 120 cm, h = 45 cm) and allowed to explore the OF for 5 min. The mouse’s behavior was recorded via an overhead Sony video camera (Tokyo, Japan). For analysis, the OF was divided into three virtual areas such as central (d = 60 cm), peripheral (r = 10 cm, near the wall), and intermediate (between central and peripheral). The following parameters were compared: distance traveled (total, central, intermediate, and peripheral), the latent period of the exit from the central area, time spent in the areas, central and intermediate area entries, and number of rearings.

#### 3.3.2. Elevated Plus Maze

The EPM is used to investigate anxiety-like behavior in rodents [29]. The test is based on the natural tendencies of rodents to avoid open or elevated places counterbalanced with their innate curiosity to explore areas that are new to them. The EPM consists of four elevated arms (two open arms, 30 × 5 × 0.5 cm; two closed arms, 30 × 5 × 15 cm), which radiate from a central platform (5 × 5 cm) forming a plus shape. Testing was carried out on the 63rd day of the experiment similarly to [17]. Briefly, a mouse was placed into the central platform facing an open arm and was allowed to explore the apparatus for 5 min. The mouse’s behavior was recorded via an overhead Sony video camera (Tokyo, Japan). The following parameters were compared: total distance traveled, the latent period of the first approach to the closed and the open arms, duration in open and closed arms, open and closed arms entries, head dipping, and the number of rearings.

#### 3.3.3. Light-Dark Box

LDB is a conventional test for the assessment of anxiety-like behavior in laboratory rodents based on the avoidance of conflict approach [30,31]. The LDB (50 × 50 × 40 cm) was divided into two parts: 1/2 of it was painted black, covered by the lid, and separated from the opened light-illuminated compartment by the wall containing an opening (8 × 6 cm) at the floor level. Testing was carried out on the 65th day of the experiment similarly to [17]. Briefly, an animal was individually placed in the middle of the lit part and allowed to explore the apparatus for 10 min. The mouse’s behavior was recorded via an overhead Sony video camera (Tokyo, Japan). Mice were compared by the following parameters: the latency to cross to the dark chamber, the time spent in the light chamber, average speed and distance traveled in the light chamber, number of times peeking out from the dark chamber, and number of transitions between compartments.

### 3.4. Silver Content Measurement in Organs

After conducting a battery of behavioral tests, the animals (n = 6–8 in each subgroup) were anesthetized with isoflurane (Baxter, Aibonito, Puerto Rico, USA) and decapitated; the organs (liver, spleen, lungs, kidneys, brain, heart, testes) and their blood were collected. This protocol was based on the widely used procedures [32,33]. Tissue samples were dried in a RedLine RF 53 drying chamber (Binder GmbH, Tuttlingen, Germany) for 72 h at 75 °C for further irradiation in the channel of a nuclear reactor. Dried samples were placed into hermetically sealed polyethylene containers at volumes of 0.2, 0.5, and 2 mL and numbered with a moisture-resistant marker (Eppendorf, Hamburg, Germany). At this time, reference samples were prepared for simultaneous irradiation in the channel of a nuclear reactor and measurements by the method of comparison with a standard sample [34]. Cotton wool was placed into the same plastic containers (to maintain the identity of the geometry factor), and a known amount of the standard sample of silver (100 or 1000 ng per sample) (LenReaktiv, Saint Petersburg, Russia) was added for this. The containers were left open, air-dried for 48 h, and then hermetically sealed. Compact reference samples were also prepared. For this, a known amount of the state standard sample of silver was placed on paper disks and air-dried. Next, plastic containers and reference samples were placed into aluminum cases made from extra pure aluminum alloy. Each aluminum case contained one reference sample with the same geometry factor as the experimental samples, as well as 84 compact samples. Aluminum cases were suspended in a vertical channel of the nuclear reactor and irradiated for 24 h at the neutron flux of 10^12^ cm^−2^ s^−1^. After irradiation, the cases were kept in the biosecurity for the decay of highly active short-lived isotopes, and after that gamma-spectrometric studies of the experimental and reference samples were carried out for the activities of the radioactive isotope ^110m^Ag with a half-life of 250 days.

### 3.5. Statistical Analysis

Data are expressed as mean ± SEM. GraphPad Prizm 6.01 software (La Jolla, San Diego, CA, USA) was used for statistical analysis, with statistical significance at *p* < 0.05. The nonparametric Kruskal–Wallis ANOVA with a post hoc Dunn’s test or Mann–Whitney U test was employed.

## 4. Results

### 4.1. Nanoparticles

**Figure 2:**
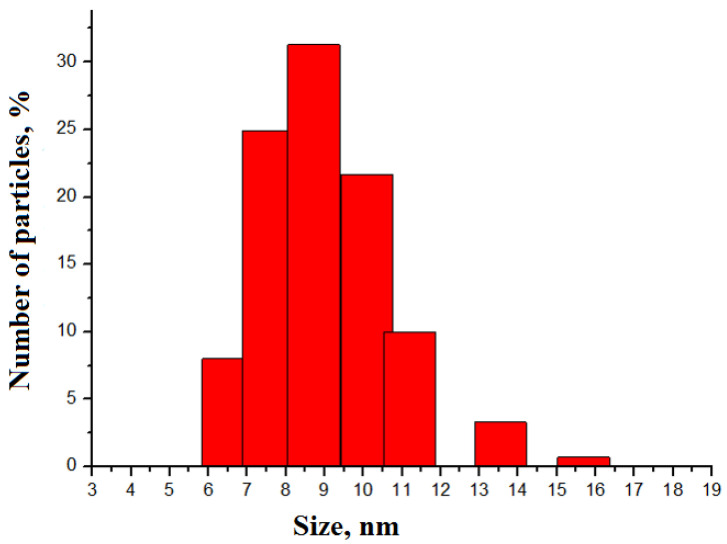
Distribution of the number of Argovit-S AgNPs by size according to the DLS data. Mean size is 8.7 ± 0.4 nm.

**Figure 3:**
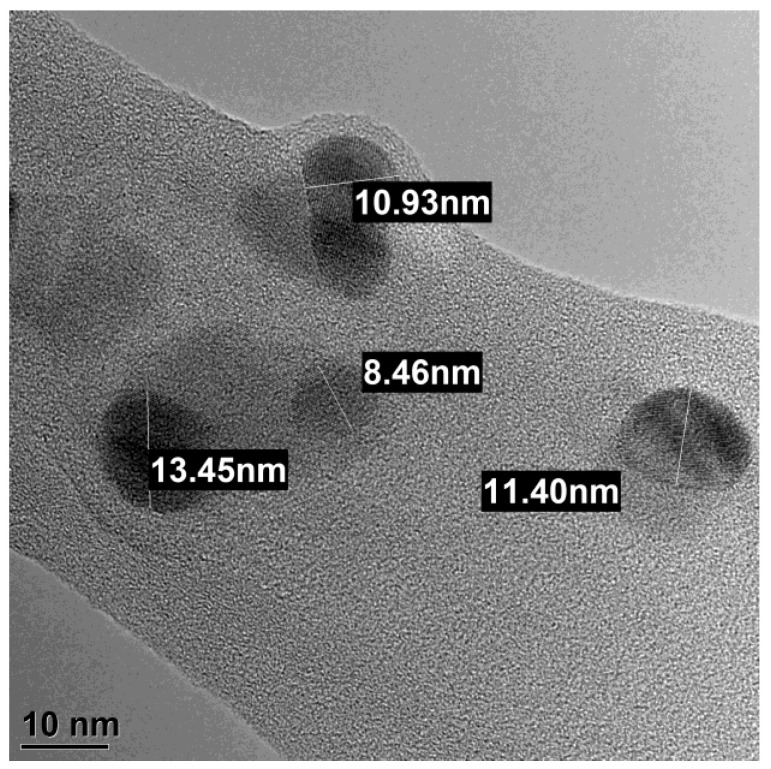
TEM image of Argovit-S AgNPs. The shape of nanoparticles is quasi-spherical.

According to DLS data, the mean size of AgNPs was 8.7 ± 0.4 nm (Figure 2). The nanoparticles were stable because their mean size did not change after storage for one year. TEM demonstrated that the shape of the nanoparticles was quasi-spherical (Figure 3). EDX has demonstrated the chemical composition of the AgNPs according to Table 1, where C was due to the carbon grid, O to the oxygen absorbed from the air, and Al to the holder material. The fraction of Ag was rather high, which proves the chemical composition of the nanoparticles under study.

### 4.2. Physiological Characteristics

**Figure 4:**
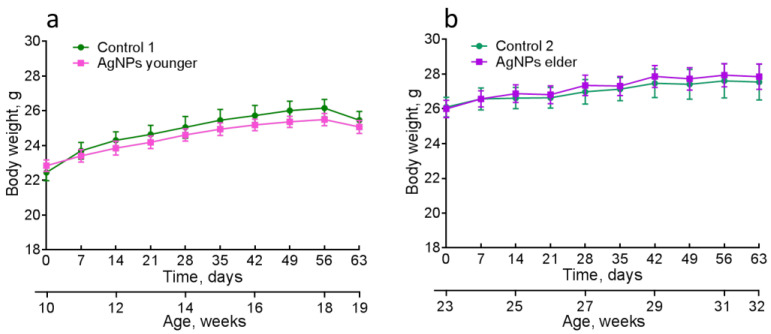
Changes in the body weight of the mice with the age: (**a**)—younger vs control 1 and (**b**)—elder vs control 2.

All the mice were gradually growing. No statistically significant difference in their body weight between the experimental and control groups was detected (Figure 4).

### 4.3. Behavioral Functions of Animals

**Figure 5:**
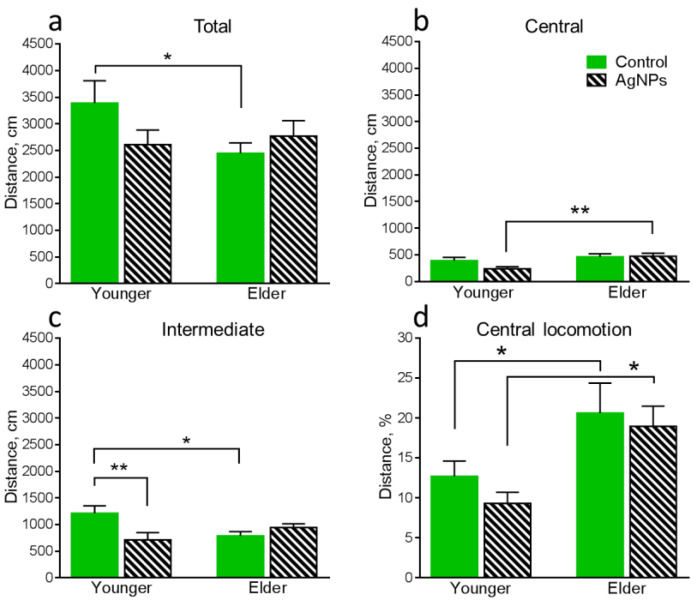
Assessment of behavioral functions in the OF: (**a**)—total distance moved in the OF, (**b**)—distance moved in the central area of the OF, (**c**)—distance moved in the intermediate area of the OF, (**d**)—central locomotion in the OF. Values are presented as mean ± SEM. * *p* < 0.05, ** *p* < 0.01.

**Figure 6:**
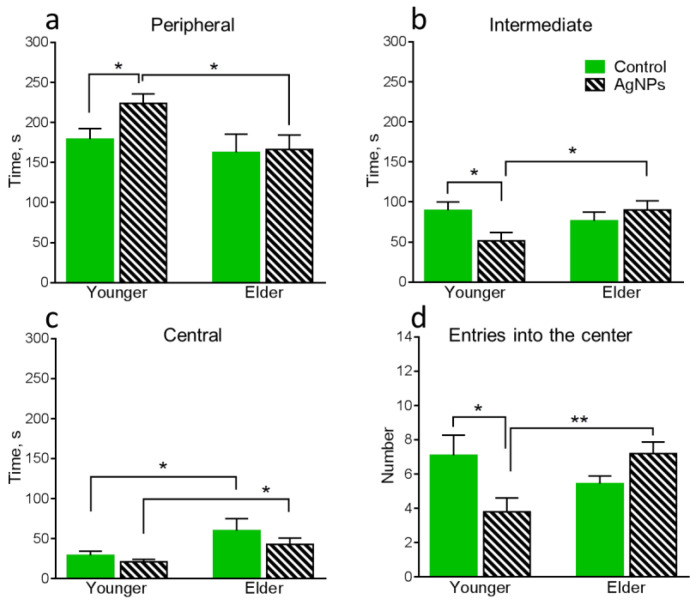
Assessment of behavioral functions in the OF: (**a**)—time spent in the peripheral area of the OF, (**b**)—time spent in the intermediate area of the OF, (**c**)—time spent in the central area of the OF, (**d**)—number of entries into the central area of the OF. Values are presented as mean ± SEM. * *p* < 0.05, ** *p* < 0.01.

**Figure 7:**
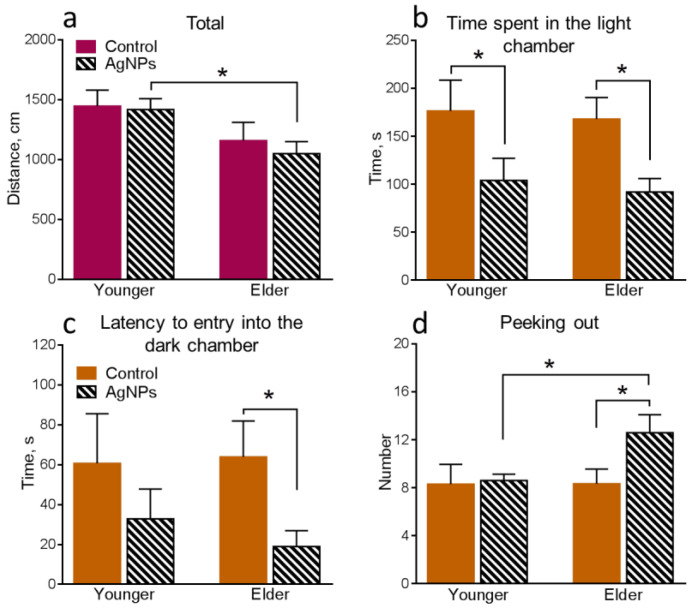
Assessment of behavioral functions in the EPM and LDB: (**a**)—total distance moved in the EPM, (**b**)—time spent in the light chamber of the LDB, (**c**)—latency to entry into the dark chamber of the LDB, (**d**)—number of times peeking out from the dark chamber of the LDB. Values are presented as mean ± SEM. * *p* < 0.05.

Statistically significantly differing results of behavioral tests for all groups of mice are present below (Figure 5, Figure 6 and Figure 7). 

Firstly, it is necessary to compare the behavioral functions of the control groups in order to detect the influence of age-related changes in the absence of exogenous perturbation in the form of AgNPs. The locomotor activity decreased with the age increase, which can be noticed from the reduction of the total distance moved (Figure 5a) and distance moved in the intermediate area (Figure 5b) of the OF. Also, anxiety simultaneously decreased with age growth, which can be seen from the increase in the time spent in the central area of the OF field (Figure 6c). 

Further, we determined the influence of oral AgNPs exposure on the younger mice in comparison with the control 1. The xenobiotic exposure significantly increases anxiety in the mice, which can be simultaneously noticed by several parameters. The distance moved in the intermediate area of the OF (Figure 5c), the time spent in this area of the OF (Figure 6b), and the number of entries into the center of the OF (Figure 6d) decreased, while the time spent in the peripheral area of the OF (Figure 6a) increased. Also, the time spent in the light chamber of LDB decreased (Figure 7b).

Behavioral functions of the elder mice exposed to AgNPs were compared with control 2. It can be seen that the mice exposed to AgNPs remain more anxious than the control ones. It can be noticed by the decrease in the time spent in the light chamber (Figure 7b) and the latency to entry into the dark chamber (Figure 7c) of the LDB. However, the number of anxiety indicators decreased in total. Also, an increase in the number of times peeking out from the dark chamber of LDB (Figure 7d) demonstrated an increase in exploration behavior, which is a positive characteristic. 

The results of the comparative analysis of the behavior of younger and elder mice both exposed to AgNPs point to a decrease in locomotor activity with age, which can be seen from the decrease in the total distance moved in the EPM (Figure 7a). A decrease in locomotor activity is typical for the elder animals in the absence of the exogenous perturbation as was shown above. A general decrease in anxiety in the elder group can be noticed in the increase in the distance moved in central (Figure 5b) and intermediate (Figure 5c) areas of the OF, a decrease in the time spent in the peripheral area of the OF (Figure 6a), as well as an increase in the number of entries into the center of the OF (Figure 6d). The elder group of mice also demonstrated an increase in exploration behavior, which was seen in the higher number of times peeking out from the dark chamber of LDB in elder animals compared with younger ones (Figure 7d). Thus, elder mice exposed to AgNPs demonstrated certain improvements in behavioral functions in comparison with the exposed younger ones. As shown above, anxiety decrease is also typical for elder control animals. Increased locomotor activity and somehow higher anxiety of younger animals can be explained by keeping them in individual cages, which is an unnatural condition for mice living in flocks in the wild nature [35]. Keeping animals in individual cages 3 months longer could neutralize the negative factor and lead to their adaptation to such stressful conditions.

### 4.4. Silver Accumulation in the Internal Organs

**Figure 8:**
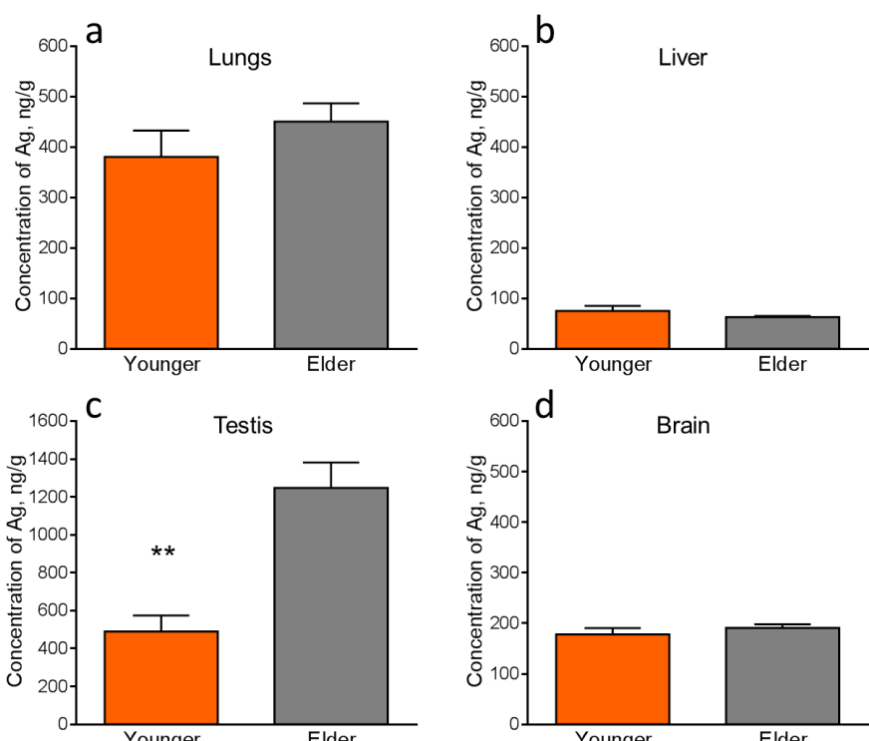
Accumulation of silver in the internal organs of younger and elder mice exposed to AgNPs: (**a**)—lungs, (**b**)—liver, (**c**)—testes, (**d**)—brain. Values are presented as mean ± SEM. ** *p* < 0.01.

**Figure 9:**
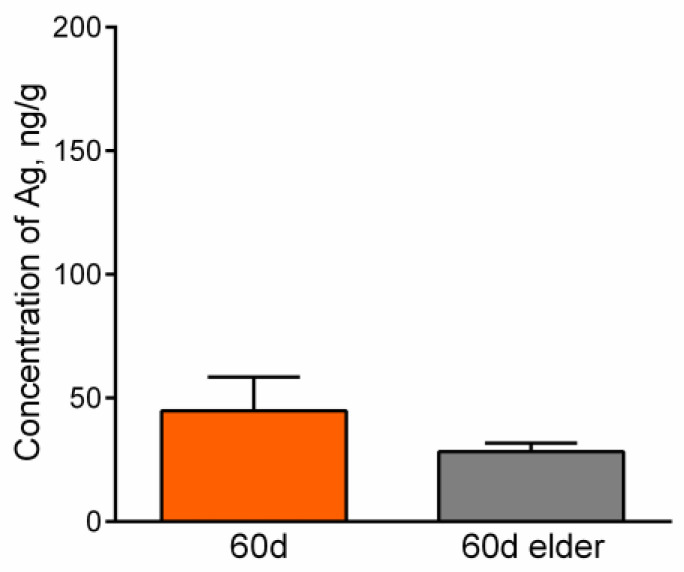
Content of silver in the kidneys of younger and elder mice exposed to AgNPs. Values are presented as mean ± SEM.

**Figure 10:**
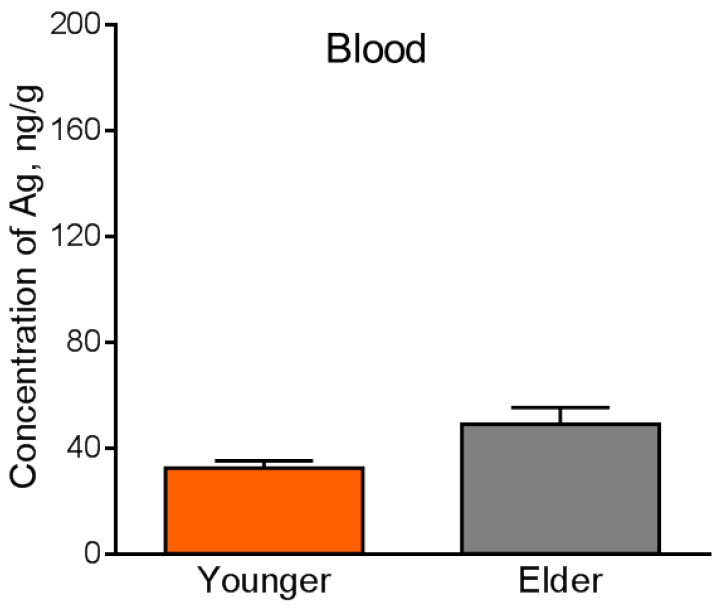
Content of silver in the blood of younger and elder mice exposed to AgNPs. Values are presented as mean ± SEM.

The content level of silver in all the control group samples was lower than the detection limit. This is because silver is not an essential element for mammalian organisms. The masses of silver in some samples of the heart and spleen were lower than the detection limit, which was an obstacle for the statistical analysis. The masses and concentrations of silver in these organs were estimated by the upper bound (Table 2). Absolute mass values of silver in the other internal organs and blood are present in Table 3. Figure 8, Figure 9 and Figure 10 show the comparative accumulation of silver in the internal organs and blood of the experimental animals with regard to age. It is seen that silver accumulates in all the considered organs and blood but in different absolute and relative values.

Figure 8 demonstrates that the level of silver accumulation in testes was statistically significantly higher in the elder than in the younger mice. It can indicate physiological changes based on the slowing down of the toxin elimination processes with age growth. Silver accumulation levels in the brain of younger and elder mice are equal. It is not higher for the elder animals. The brain is the crucial organ for behavioral functions and hypothetically, their disturbance might be associated with the accumulation of potential xenobiotics.

Significant concentrations of silver are detected in the testes, lungs, and brain in all exposed groups, which may be due to the specificity of the blood-organ barriers in them. Concentrations of silver in the liver and kidneys for both ages were relatively low (Figure 8 and Figure 9), which can be caused by the anatomical and physiological peculiarities of these organs and their role in the organism to cleanse and neutralize toxins. Relatively low concentrations and masses of silver are found in the blood (Figure 10). The low content of silver in the blood is typical for subacute, subchronic, and chronic exposures to AgNPs [16] and corresponds to static equilibrium. A high content of silver in the blood is observed at acute exposure to AgNPs [36]. Such redistribution is caused by the transport function of this biological liquid. It has already delivered the largest fraction of the administered exogenous substance to tissues by the observed period of time. Relatively low concentrations of silver in the spleen (Table 1) are also due to the anatomic and physiological peculiarities of the lymphatic system, of which this organ is a part. Absorption and transport of silver are implemented into and by the blood, which brings it into the spleen. Only the efferent lymphatics are connected to the spleen and exogenous substances may enter it only with the blood supply, presuming, in the composition of blood cells or plasma proteins. The spleen, as a part of the lymphatic system, cleans the organism from pathogens by activation of T- and B- lymphocytes. A certain part of silver may be stuck in the spleen; however, it does not possess the mechanisms for neutralization of such inorganic compounds. The smallest concentration of silver was observed in the heart (Table 1), which is caused by its functions providing high metabolism, fast clearance, and renewal by the intensive bloodstream. Absolute mass values of silver in the heart and spleen are also low (Table 1). We should note that INAA does not show the form and shape of silver accumulated in the organs. This point should be investigated further by the application of Electron Microscopy.

## 5. Discussion

Silver accumulates in the organs of the animals orally exposed to AgNPs and, in particular, its accumulation in the brain is equal for the younger and elder mice. The exposed animals of both ages demonstrated anxiety increase, however, in varying degrees. It was suggested in [20] that a disturbance of behavioral functions in mammals exposed to AgNPs is due to the accumulation of silver in the brain and the oxidative stress induced in it by reactive oxygen species generated by the silver compound. The elder mice in comparison with the younger ones demonstrated a certain decrease in anxiety. The xenobiotic in the form of AgNPs showed a hormetic effect [2] manifested in the increase of exploration behavior in the elder animals and better adaptation to the xenobiotic related to the improvement of adaptive homeostasis in them. It correlates with the concept that improvement of physiological functions and adaptive homeostasis takes place until 1/3 of the maximal lifespan. Herewith, it is likely that physiological and cognitive functions are related in direct proportion. It was shown earlier that physiological and cognitive functions are associated [37], i.e., mental health and physiology are inextricably linked. Good physical characteristics correlate with good cognitive abilities [38]. Also, it is known that cognitive functions improve until middle age, which is confirmed by the higher IQ levels of 35–40 years old humans in comparison with other age groups [39]. Cognitive processes have been theorized to be among the mechanisms that drive behavior [40], and it is likely that good cognitive functions correlate with good behavioral functions and vice versa. A correlation between cognition and behavior was found in epilepsy [41]. Thus, mental and physiological functions are the facets of the same crystal, and a good quality of adaptive homeostasis is directly proportional to good physiology and cognition. 

We observed a neurotoxicity decrease of AgNPs with aging manifested in the improvement of certain behavioral functions of elder animals in comparison with younger ones. This indicated the increase of adaptive homeostasis quality with age in the period of vitality blossom. Herewith, we observed more significant silver accumulation in the testes of elder animals compared with that in younger ones. Presumably, higher silver accumulation in the testes may cause other toxic effects in the reproductive system, which were not considered in the present work, but in [11,22,42,43,44]. Exposure to AgNPs may influence mice fertility [45]. Also, AgNPs may be transported from the exposed during pregnancy females to the offspring via placental and mammary gland barriers [46]. Thus, transited AgNPs, silver ions, and granules may accumulate in the offspring’s organs and cause toxic effects on them. Reproductive system toxicity in regard to age is the point of future research.

Also, bioaccumulation of the potential xenobiotic may cause toxic effects later, after the beginning of the decline of adaptive homeostasis and weakening of its function as well. We observed a decline in long-term contextual memory in mice after 180 days of exposure to AgNPs of the same trade mark caused by silver accumulation in the brain and its departments [17,20]. Besides direct toxic effects in the tissues where AgNPs accumulate, they can exert toxicity at the whole organism level, which can be explained within the framework of the neurovisceral integration concept [47]. Within the concept of neurovisceral integration, toxicity in different organs may lead to a disturbance in the function of certain hemispheres [48], and dysfunctions in the brain may lead to a broad range of dysfunctions in the organism. 

Returning to the work’s objective and summarizing the above-mentioned, it can be concluded that age growth is not directly conjugated with the organism fading and pathology development. Oppositely, vitality and resistance to xenobiotics may even improve with age at least until the prime of life. A pathology development in a human is presumably caused by some other more complex mechanisms of a psycho-somatic nature [49]. The growth of age enhances the probability of such mechanisms’ implementation but is not the direct reason for pathology development and death.

## 6. Conclusions

There are two contradictory concepts about adaptive homeostasis development with age. The first one states that it proportionally declines to age growth. The second concept affirms that adaptive homeostasis non-linearly changes during the life span. It increases with age during vitality blossom and starts to decline from 1/3 of the maximal lifespan. 

In the present work, we observed the change in the behavioral functions of laboratory mice as a mammalian model under the influence of a xenobiotic in the form of AgNPs with regard to age. Elder animals demonstrated improvement in behavioral functions in comparison with younger ones despite the increase of the accumulated silver in the testes. The levels of silver accumulation in the brain, lungs, liver, kidneys, and blood were equal for both ages. They were the highest in the brain, testes, and lungs. Accumulation of xenobiotics in the heart and spleen was low. The observed phenomenon can be explained by the better adaptation of the animals to the potential xenobiotic in elder age. Adaptive homeostasis non-linearly changes with age. Thus, our work confirms the second concept rather than the first one. Vitality and resistance to xenobiotics improve at least until the prime of life.

## Figures and Tables

**Table 1:** Chemical composition of the Argovit S AgNPs obtained by EDX.

No.	Element	Ratio
1	C	0.62
2	O	0.0165
3	Al	0.0006
4	Ag	0.039

**Table 2:** Concentration of silver in the internal organs of the mice exposed to AgNPs. Values are performed as mean.

Organ	Group	Concentration, ng/g	Mass, ng
Heart	Younger	<11.2	<1.7
	Elder	<15	<2.4
Spleen	Younger	<24.6	<1.5
	Elder	<76.4	<4.6

**Table 3:** Absolute content of Ag in the mice’s internal organs and blood. Values are performed as mean ± SEM.

Mass, ng						
Organ	Brain	Testes	Lungs	Liver	Kidneys	Blood
Younger	85 ± 19	88 ± 31.5	78 ± 34	102 ± 41	18 ± 8	20 ± 4
Elder	82 ± 5	219 ± 58.5	97 ± 9	98 ± 13	12 ± 1	37 ± 12

## Data Availability

Not applicable.
